# Complex Molecular Evolution and Expression of Expansin Gene Families in Three Basic Diploid Species of *Brassica*

**DOI:** 10.3390/ijms21103424

**Published:** 2020-05-12

**Authors:** Weimiao Liu, Tianqi Lyu, Liai Xu, Ziwei Hu, Xingpeng Xiong, Tingting Liu, Jiashu Cao

**Affiliations:** 1 Laboratory of Cell and Molecular Biology, Institute of Vegetable Science, Zhejiang University, Hangzhou 310058, China; 11616044@zju.edu.cn (W.L.); 11716015@zju.edu.cn (T.L.); xuyuanchicao@163.com (L.X.); 11416051@zju.edu.cn (Z.H.); xiongxingpeng1989@163.com (X.X.); 11416009@zju.edu.cn (T.L.); 2 Key Laboratory of Horticultural Plant Growth, Development and Quality Improvement, Ministry of Agriculture, Hangzhou 310058, China; 3 Zhejiang Provincial Key Laboratory of Horticultural Plant Integrative Biology, Hangzhou 310058, China

**Keywords:** *Brassica* species, expansin gene family, molecular evolution, promoter divergence, expression pattern, reproductive development

## Abstract

Expansins are a kind of structural proteins of the plant cell wall, and they enlarge cells by loosening the cell walls. Therefore, expansins are involved in many growth and development processes. The complete genomic sequences of *Brassica rapa*, *Brassica oleracea* and *Brassica nigra* provide effective platforms for researchers to study expansin genes, and can be compared with analogues in *Arabidopsis thaliana*. This study identified and characterized expansin families in *B. rapa*, *B. oleracea*, and *B. nigra*. Through the comparative analysis of phylogeny, gene structure, and physicochemical properties, the expansin families were divided into four subfamilies, and then their expansion patterns and evolution details were explored accordingly. Results showed that after the three species underwent independent evolution following their separation from *A. thaliana*, the expansin families in the three species had increased similarities but fewer divergences. By searching divergences of promoters and coding sequences, significant positive correlations were revealed among orthologs in *A. thaliana* and the three basic species. Subsequently, differential expressions indicated extensive functional divergences in the expansin families of the three species, especially in reproductive development. Hence, these results support the molecular evolution of basic *Brassica* species, potential functions of these genes, and genetic improvement of related crops.

## 1. Introduction

The cell wall is one of the main differences between plant cells and animal cells, and it is a highly dynamic and complex network, which is closely related to many processes of plant growth and development. The size and shape of various plant organs are precisely controlled through regional cell division, and subsequent cell expansion during plant growth [1]. While inhibiting the expansion of protoplasts, the cell wall undergoes stress relaxation due to various factors. The cells are always in a dynamic equilibrium of protoplast expansion and cell wall restraint, and undergo irreversible expansion [2]. The enlargement of the cell walls begins with the stress relaxation of the walls, which enables the cells to absorb water and physically enlarge. Expansins are a class of nonenzymatic wall proteins. They mediate ‘acid-induced growth’ by disrupting the non-covalent linkages that hold microfibrils in place in the cell wall. [3].

Expansins are encoded by a multigene family, that induces pH-dependent cell wall expansion and stress relaxation in a characteristic and unique manner [4]. They are typically 250–275 amino acids long and are made up of two domains (DPBB_1 and Pollen_allerg_1) that are taken over the lead by a signal peptide [5]. According to phylogenetic and conserved sequence analyses, the expansin family has been classified into four subfamilies, namely, EXPA, EXPB, EXLA, and EXLB [6]. Members of the EXPA and EXPB subfamilies are involved in most plant growth and developmental processes, whereas the functions of EXLA and EXLB proteins are unclear [2].

Expansins play important roles in a variety of biological processes related to cell wall modification. In general, different subfamilies assume different responsibilities. For example, the EXPA subfamily plays a role in seed germination [7], root elongation [8,9], leaf growth [10], and fruit maturation and softening [11], whereas the EXPB subfamily plays a part in pollen germination and fertilization [12,13,14]. Expansins are also involved in plant–environment interactions, including responding to a variety of abiotic stresses [15,16,17,18] and interacting with exogenous stimuli such as pathogens [19,20,21]. In addition, these studies found a unique relationship between the EXPB subfamily with the process of pollen development. However, related studies are limited at present, and the specific regulatory mechanism is still unclear. Therefore, further studies on the EXPB subfamily and exploration of the expansin genes related to pollen development will provide insights into the important processes of plant reproductive development. Expansins have also been reported in many plant species and organs, including *Micrasterias denticulata* [22]; *Physcomitrella patens* [23]; *Pinus taeda*, *A. thaliana*, and rice [5]; maize [24]; soybean [25]; tobacco [26]; and apple [27]. They are all shown in Table 1. Although a previous study reported the profile of the expansin family in *B. rapa*, the characteristics of their evolutionary destiny can still be observed by comparing the expansin families in the three basic *Brassica* species [28].

Brassiceae includes the cultivated *Brassica* species, which are a group of crops that are most closely related to *A. thaliana* [29]. *Brassica* and *A. thaliana*, which belong to the mustard family Brassicaceae, are an exemplary plant and crop, respectively [30]. Approximately 20 million years ago (MYA), *Brassica* and *A. thaliana* were diverged from a common ancestor; then, approximately 16 MYA, *Brassica* ancestors underwent a whole genome triplication (WGT) event [31], and phylogenetic analysis divided the species of *Brassica* into *B. rapa*, *B. oleracea*, and *B. nigra* lineages, which diverged approximately 8 MYA [32]. In *Brassica*, the interspecific cytogenetic relationship between important crops is well described with a “U” triangle, with two diploid species each (*B. rapa* (A, n = 10), *B. oleracea* (C, n = 9), and *B. nigra* (B, n = 8)) forming a tetraploidy species (*B. napus* (AC, n = 19), *B. juncea* (AB, n = 18), or *B. carinata* (BC, n = 17)) [33]. The genus *Brassica* offers an opportunity to study changes in expansin families through comparative genomics with the model plant *A. thaliana*.

In this study, the genome-wide identifications and phylogenetic analyses of the expansin genes were conducted in *B. rapa* (*BrEXPs*), *B. oleracea* (*BolEXPs*), and *B. nigra* (*BniEXPs*). The structure of exon–intron, conserved motifs and domains, chromosomal localization, synteny, retention rates, selection pressures, and coding sequence and promoter evolution were explored. The expression patterns of *B. rapa*, *B. oleracea*, and *B. nigra*, especially in the process of reproductive development, were also investigated. The relationship between promoter variation and coding sequence was also characterized. Horizontally, our work provides several clues to understand the similarities and differences in the genomes of the three basic species of *Brassica*. Vertically, by introducing *A. thaliana* as a reference, several simple guesses can also be made about the process of evolution among the three basic species.

## 2. Results

### 2.1. Genome-Wide Identification of the Expansin Gene Family in Three Basic Diploid Species of Brassica

With the three-step analysis, 56, 58, and 60 expansin genes were identified in *B. rapa*, *B. oleracea*, and *B. nigra*, respectively (Table 1). All members contained two domains, namely, DPBB_1 and Pollen_allerg_1, on the basis of Pfam, SMART, and CDD/SPARCLE tests. Proteins that had only one of these domains or did not have an integral open reading frame were excluded after reannotation by the FGENESH tools of the Softberry website. Compared with a previous study [28], we reannotated *Bra000142*, *Bra004891*, and *Bra016981* and identified three more *BrEXPs*. At the same time, 11 *BolEXPs* and 8 *BniEXPs* were also reconfirmed (Table S2). Particularly, *BniB012123* was reannotated into two genes, both of which had the complete structure of the expansin genes, and were renamed *BniB012123-1* and *BniB012123-2*.

The expansin gene family is a large family with four subfamilies, namely, EXPA, EXPB, EXLA, and EXLB. To clarify which subfamily these expansin genes belong to, we employed MEGA 6.0 to construct a phylogenetic tree with the maximum likelihood algorithm using the entire expansin protein sequences of *A. thaliana*, *B. rapa*, *B. oleracea*, and *B. nigra*. Given that the expansin genes of *A. thaliana* have already been classified, we were able to classify the expansin genes according to the clustering exhibited on the phylogenetic tree. The number of genes in each subfamily is shown in Table 1. On the basis of the nomenclature rules proposed by a previous study [6], we named the expansin genes in *B. rapa*, *B. oleracea*, and *B. nigra* using their genome locations and the subfamily to which they belonged (Table S2).

**Figure 1:**
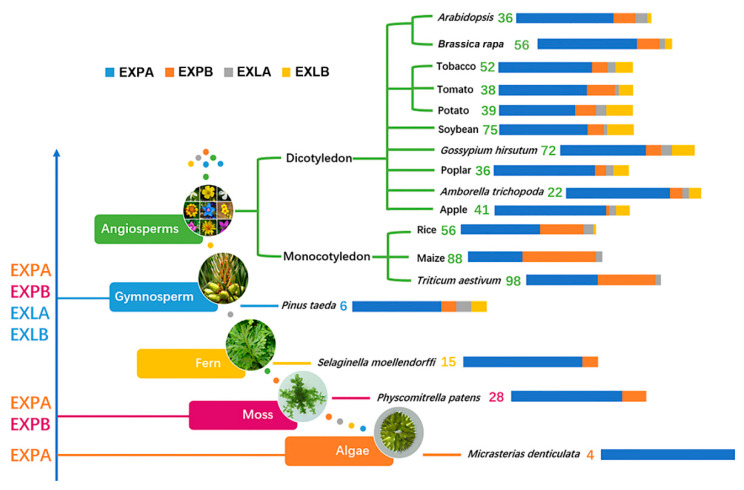
Evolutionary analyses of expansin genes, numbers of family members, and proportions of different subfamilies in representing species. Different subfamilies emerged at different periods during evolution, with appearance times indicated by blue arrows on the left. Expansin family members are shown for different species, with changes in the proportions of different subfamilies. Different bar lengths represent different proportions of subfamilies. The numbers of family members in representative species are from reported studies. References are the same as in Table 1.

We compared the number of expansin genes in the three basic diploid species of *Brassica* to other available genomes and found that EXPA and EXPB subfamilies accounted for the largest proportion of the entire family and appeared first in the evolutionary process of plants (Figure 1). The EXPB subfamilies in monocotyledonous plants were also larger than the dicotyledonous plants, whereas those of the three basic species of *Brassica* were similar in size to *A. thaliana* and larger than other dicotyledons currently known. Although *B. rapa*, *B. oleracea* and *B. nigra* had more expansin genes than *A. thaliana*, none of them had tripled that of *A. thaliana*. This result indicated that approximately 50% of *BrEXPs*, *BolEXPs*, and *BniEXPs* had been deleted during the evolution process after WGT (Table 1).

A detailed characterization of the these expansin genes, including gene names, gene IDs, positions, predicted amino acids, isoelectric points (pIs), molecular weights (Mw), and signal peptide positions, is provided in Table S2. The isolated BrEXPs encode proteins ranging from 170 to 360 amino acids in size with an average length of 259 amino acids and a molecular weight ranging from 17.9 to 40.9 kD, except BrEXPA20 (Br016981), which contained not only DPBB_1 and Pollen_allege_1 but also two different macro domains, namely, PF01661 and PF01283. The 58 and 60 expansins in *B. oleracea* and *B. nigra* were 141–342 and 190–372 amino acids long, with molecular weights ranging from 14.9 kD to 38.5 kD and 19.9 kD to 42.2 kD, respectively. Most BrEXPs, BolEXPs, and BniEXPs contained signal peptides with a length of 17 to 30 amino acids, except for 7 BrEXPs, 8 BolEXPs, and 2 BniEXPs. The pI values ranged from 4.52 to 10.74 in *A. thaliana* and three basic species of *Brassica*. These characteristics of the EXLB subfamily in the four species were very conserved. Among them, the average of pI values was less than 7.0 in the EXLB subfamily and above 7.0 in other subfamilies. Meanwhile, the lengths of the signal peptides and proteins of the EXLB subfamily were the same, except for BolEXLB1, which was 251 amino acids (Table S2).

### 2.2. Phylogenetic and Structural Analyses of BrEXPs, BolEXPs, and BniEXPs

**Figure 2:**
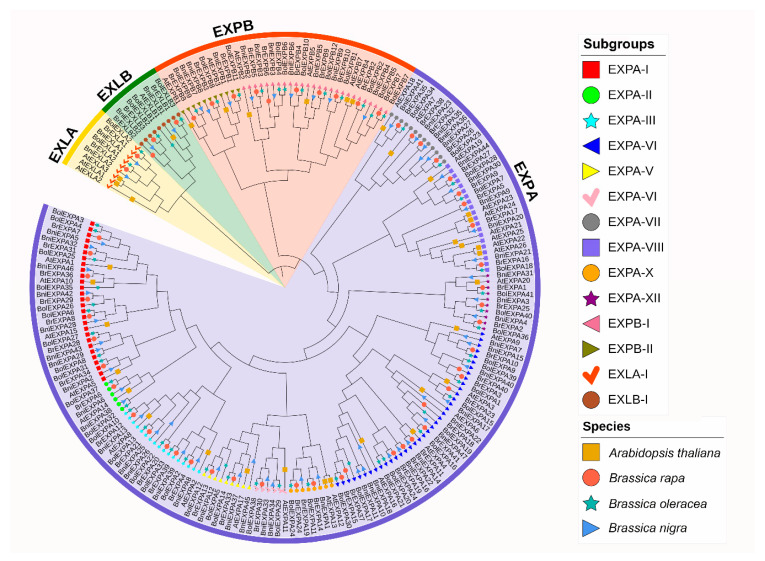
Phylogenetic relationship of *A. thaliana*, *B. rapa*, *B. oleracea*, and *B. nigra* expansin genes. The phylogenetic tree was constructed based on the complete protein sequence alignment of expansins in the four species by the maximum likelihood algorithm with bootstrapping analysis (1000 replicates) and the ‘WAG + G’ model. The four subfamilies are marked by colorful background.

The phylogenetic tree of expansin genes in the three basic diploid species of *Brassica* and *A. thaliana* was constructed based on their deduced amino acid sequences from the multiple sequence alignment. Phylogenetic analysis showed that members of four subfamilies were clustered together according to their evolutionary relationships (Figure 2).

**Figure 3:**
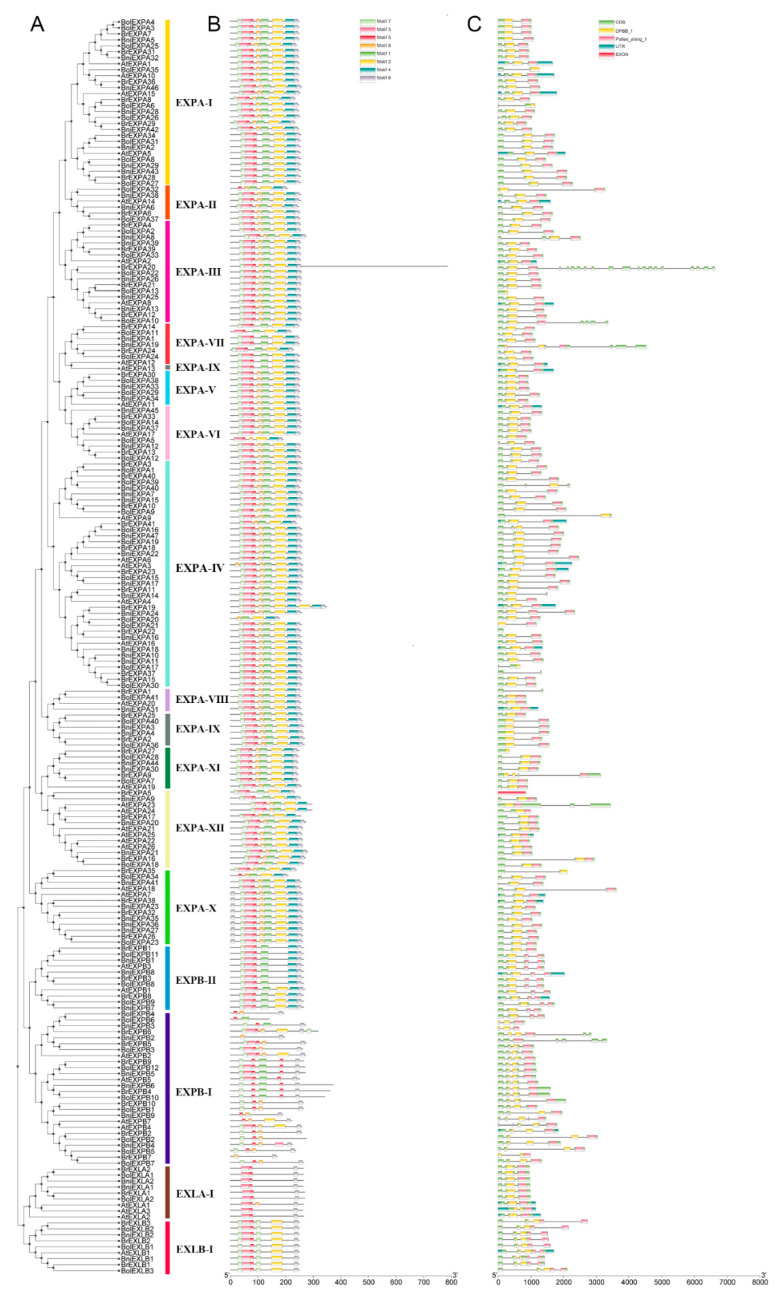
Phylogenetic relationships and classification of subgroups (**A**), conserved motifs analyses (**B**), gene structure and domain distribution (**C**) of expansin genes in *A. thaliana*, *B. rapa*, *B. oleracea*, and *B. nigra*. The phylogenetic tree constructed with MEGA 6.0, different subgroups were marked by bars with different colors. The motif compositions were detected with an online tool, MEME, which 8 different colored boxes were found in expansin genes. Different gene structures and domain distribution were represented by different colored boxes whereas introns were shown in gray lines. The scale underneath was to measure amino acids (**B**) or nucleotides lengths (**C**).

According to the grouping methods used for *A. thaliana* and rice expansin gene families [5] and phylogenetic tree clustering, we divided expansin genes of the four species into EXPA with 12 subgroups, EXPB with 2 subgroups, and EXLA and EXLB with 1 subgroup. These subfamilies were sorted in Roman alphabetical order. *AtEXPA19* was classified as EXPA-XI, which showed a different concept from the previous definition of EXPA-XI as a specific subgroup in rice [5]. Among these subgroups, EXPA-IV was the largest clade with 38 *EXPAs*, which was consistent with the research in tobacco [26], whereas EXPA-VIII was the smallest clade and contained only four *EXPAs* (Figure 3).

**Figure 4:**
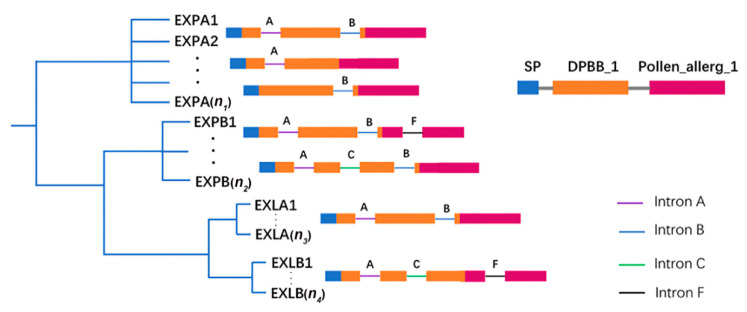
The gene structure and domain model of the four expansin subfamilies. Usually *n_1_* is the largest, followed by *n_2_*, *n_3_*, and *n_4_*. SP, signal peptide, DPBB_1 and Pollen_allerg_1 are two typical domains of expansins. Intron A, B, C and F were obtained from [5].

To obtain additional insights into the possible gene structural evolution and provide valuable information concerning duplication events when interpreting phylogenetic relationships within gene families, the exon–intron structures of expansin family genes in *A. thaliana*, *B. rapa*, *B. oleracea* and *B. nigra* were analyzed (Figure 3C). The number of introns varied from 0 to 5, except for *BrEXPA20*. Basically, expansin genes in the same subfamily had similar exon–intron structures. In accordance with ancient exon–intron pattern, the introns of the expansin genes were named A, C, B, F, H, and I [5,28]. Most expansin genes in the EXPA subfamily contained two introns, A or B, or only one of A and B. However, most members of the EXPB subfamily belonged to two exon–intron structural patterns, namely, A-B-F and A-C-B. In particular, the members of the EXLA subfamily contained both A and B introns, whereas those of the EXLB subfamily had A, C, and F introns (Figure 4). A previous study reported that most of the genes encoding EXLA and EXLB proteins belong to the exon–intron structure pattern of A-C-B-F, which was inconsistent with our conclusion [5]. This result showed that unique exon–intron patterns were present in the EXLA and EXLB subfamilies of the four species.

### 2.3. Chromosomal Distribution, Duplication Mechanism and Retained Proportion in Three Basic Species of Brassica

**Figure 5:**
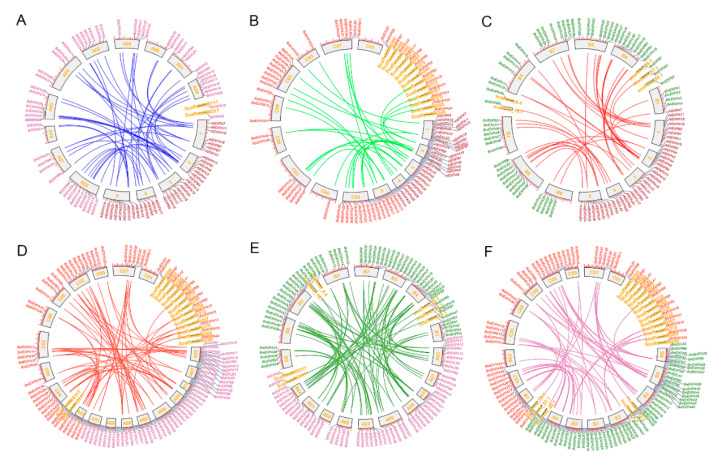
The syntenic analyses and positions on corresponding genomes of expansin genes in the four species. (**A**–**C**) The orthologous gene pairs between *A. thaliana* and *B. rapa* (**A**), *A. thaliana* and *B. oleracea* (**B**), and *A. thaliana* and *B. nigra* (**C**). (**D**,**E**) The homologous gene pairs between *B. rapa* and *B. oleracea* (**D**), and *B. rapa* and *B. nigra* (**E**). (**F**) The homologous gene pairs between *B. oleracea* and *B. nigra.*

To explore the underlying duplication mechanism accounting for the expansion of the expansin gene family, we explored the genomic distributions of the expansin genes in three basic species. Except for 2 *BrEXPs*, 14 *BolEXPs*, and 5 *BniEXPs*, 54, 44, and 55 expansin genes were located on the chromosomes of *B. rapa*, *B. oleracea*, and *B. nigra*, respectively. Except for the C01 chromosome of *B. oleracea*, every other chromosome had uneven distribution of expansin genes (Figure S1). In *B. rapa*, *B. oleracea*, and *B. nigra* genomes, 12.5%, 22.4%, and 15% expansin genes were formed by tandem duplication and distributed in 3, 5, and 4 tandem arrays of 2–5 genes, respectively. However, this ratio was up to 27.8% in *A. thaliana*. (Figure S1). Using *A. thaliana* as a reference, we also traced these orthologous expansin gene pairs between *A. thaliana* and *Brassica* species to detect their evolutionary history. From the analysis of the orthologous regions for comparative analysis, we obtained 49, 29, and 39 sets of orthologous genes between *A. thaliana* and *B. rapa*, *A. thaliana* and *B. oleracea*, and *A. thaliana* and *B. nigra*, as shown in Figure 5. In addition, we performed collinearity analyses of expansin genes among the three *Brassica* species and their individual genomes. We acquired 67 homologous gene pairs between *B. rapa* and *B. oleracea*, 88 between *B. rapa* and *B. nigra* and 56 between *B. oleracea* and *B. nigra* (Figure 5). These results indicated that segmental duplications played important roles in expansin gene family expansion in *Brassica* genomes.

**Figure 6:**
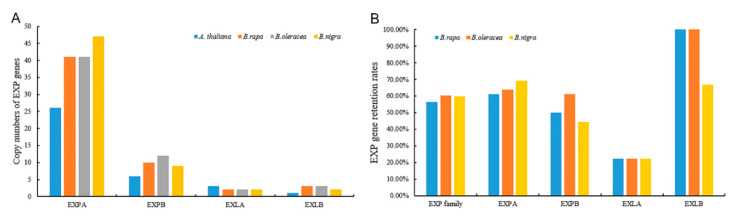
Copy numbers (**A**) and gene retention rates (**B**) of expansin genes in the four subfamilies and the three basic species of *Brassica*.

Further analyses on expansin genes’ retention in *Brassica* species after WGT were performed. On the one hand, EXPA subfamily genes occupied the major part of the entire family in each basic species of *Brassica* (Figure 6A). On the other hand, the retention rates of the EXPA and EXLB subfamilies in three species significantly exceeded the overall rate of the corresponding family, whereas those of the EXLA subfamilies were lower (Figure 6B).

### 2.4. Coding Sequence and Promoter Evolution Analyses of Expansin Genes in the Three Species of Brassica

To understand the evolution of coding sequences of different branch members of expansin families in the three species of *Brassica*, we calculated the ω ratios (the number of nonsynonymous substitutions per nonsynonymous site (*Ka*)/the number of synonymous substitutions per synonymous site (*Ks*)) for different subfamilies of expansin genes with *A. thaliana* as the reference (Table 2). For *BrEXPs*, *BolEXPs*, and *BniEXPs*, the *LRT p*-values between one and free-ratio models were higher than 0.05. This result indicated that no significant differences were among ω values in different expansin subfamilies. Their ω ratios were also between 0 and 1, indicating that they were undergoing purification selection. EXLB subfamilies also had the smallest ω values, suggesting that their sequences were the most conserved, which was also consistent with the results of previous physical and chemical analyses. Afterward, we calculated the ω ratio of each subgroup in the three species and found that only a few subgroups (EXPA-IV and EXPB-II for *B. rapa*, EXPA-IV fo*r B. oleracea* and EXPA-XI for *B. nigra*) had significant differences (Table S5).

**Figure 7:**
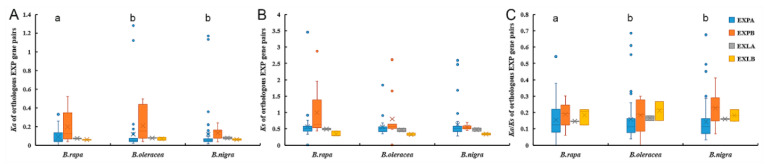
The *Ka*, *Ks*, *Ka*/*Ks* distributions of orthologous expansin gene pairs between *A. thaliana* and *B. rapa*, *A. thaliana* and *B. oleracea*, and *A. thaliana* and *B. nigra.* (**A**–**C**) *Box plots* of *Ka*, *Ks*, and *Ka*/*Ks* of orthologs. Vertical lines represent the range of values, boxes represent interquartile distances, and the colored line represents the median. The circles represent the abnormal values and the crosses represent the average values. Lower-case letters (a and b) indicate significant differences (*p* < 0.05, Tukey test).

The *Ka* and *Ks* substitution rates and *Ka*/*Ks* ratios for orthologous gene pairs were used to detect the evolutionary selection pattern of expansin genes among the four species (Figure 7, Table S3). According to distributions, several “abnormal” expansins were observed in several subfamilies, such as *BrEXPA33* and *BrEXPA13*, which showed *Ka* and *Ks* of up to 0.126 and 1.070, respectively. Without considering abnormal values, in EXPA subfamilies, the orthologous *Ka* and *Ka*/*Ks* values between *B. rapa* and *B. oleracea*, and between *B. rapa* and *B. nigra* were significantly different, but there was no significant difference between *B. oleracea* and *B. nigra*. In other subfamilies, the differences among the three species were not significant, either. The sequences of BrEXPAs exhibited the largest sequence changes, whereas BniEXPAs were highly conserved, with mean *Ka* and *Ka*/*Ks* values as low as 0.048 and 0.114, respectively (Table 3). For the *Ks* values of the expansin gene pairs in the three species, the discrepancies among species or subfamilies were not significant.

**Figure 8:**
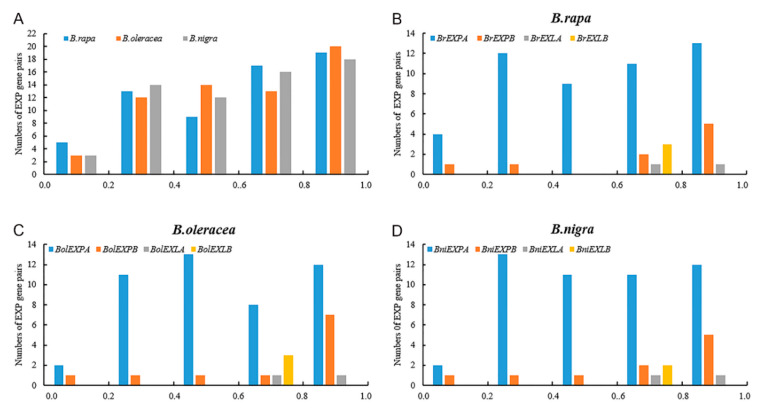
The *d_SM_* distributions of orthologous expansin gene pairs between *A. thaliana* and *B. rapa*, *A. thaliana* and *B. oleracea*, and *A. thaliana* and *B. nigra*. (**A**) The *d_SM_* distribution in the three basic species of *Brassica*. (**B**–**D**) The *d_SM_* distribution in the four subfamilies of *B. rapa*, *B. oleracea*, and *B. nigra*.

The promoter divergence of the orthologs were determined by *SharMot*. At the species level, we found that the distribution of expansin gene pairs in the three species increased with the growth of *d_SM_* values and the peak value of *d_SM_* ranged from 0.8 to 1.0, indicating that the degree of promoter divergence was considerably high (Figure 8A). At the subfamily level, EXPA subfamilies had the least distribution between 0 and 0.2, and it was evenly distributed in the remaining intervals. However, the three other subfamilies were mainly distributed between 0.6 and 1.0, especially for EXLA and EXLB subfamilies (Figure 8B–D, Table 3), demonstrating that the promoters of the two subfamilies showed a higher overall divergence during the evolutionary process. For the relatively limited number of paralogs, we found that the distribution of *d_SM_* values was relatively uniform, both at the species and family level. However, among the three species, the *Ka*/*Ks* values of the EXPA subfamilies were the smallest among the subfamilies, indicating that their coding sequences were the most conservative (Figures S2 and S3, Tables S4 and S6).

**Figure 9:**
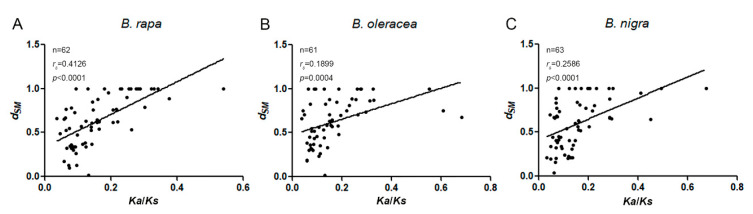
Correlation between *d_SM_* and *Ka*/*Ks* of orthologous expansin gene pairs between *A. thaliana* and *B. rapa* (**A**), *A. thaliana* and *B. oleracea* (**B**), and *A. thaliana* and *B. nigra* (**C**). We found significant positive correlations between *Ka*/*Ks* and shared motif divergence in sequences 0–500 bp upstream of translation start of orthologues (Pearson correlation). Linear fits of these data are also plotted (A: *y* = 0.336 + 1.856*x*; B: *y* = 0.479 + 0.879*x*; C: *y* = 0.410 + 1.199*x*).

To explore whether a correlation exists between the promoter and coding sequence evolution of the expansin family members, we performed a multiple regression analysis involving *Ka*, *Ks*, *Ka*/*Ks*, and *d_SM_*. We observed weak but extremely significant correlations between *d_SM_* and Ka/Ks (*d_SM_* vs. (*Ka*/*K*s), *r_s_* ≤ 0.42, *p* ≤ 0.0004; multiple regression formula: *d_SM_*~*Ka*+*Ks*+(*Ka*/*Ks*), Pearson correlation) between *A. thaliana* and *B. rapa*, *A. thaliana* and *B. oleracea*, and *A. thaliana* and *B. nigra* (Figure 9). This result indicated that when the promoter sequence of an expansin gene had a large variation, its corresponding coding sequence was also less conservative. However, no significant correlations were detected between *d_SM_* and *Ka*/*Ks* in the paralogs of *B. rapa*, *B. oleracea*, and *B. nigra* (multiple regression formula: *d_SM_*~*Ka* + *Ks* + (*Ka*/*Ks*)) (Figures S2 and S3).

### 2.5. Expression Patterns of Expansin Family Members in Various Tissues and Organs of B. rapa, B. nigra, and B. oleracea

**Figure 10:**
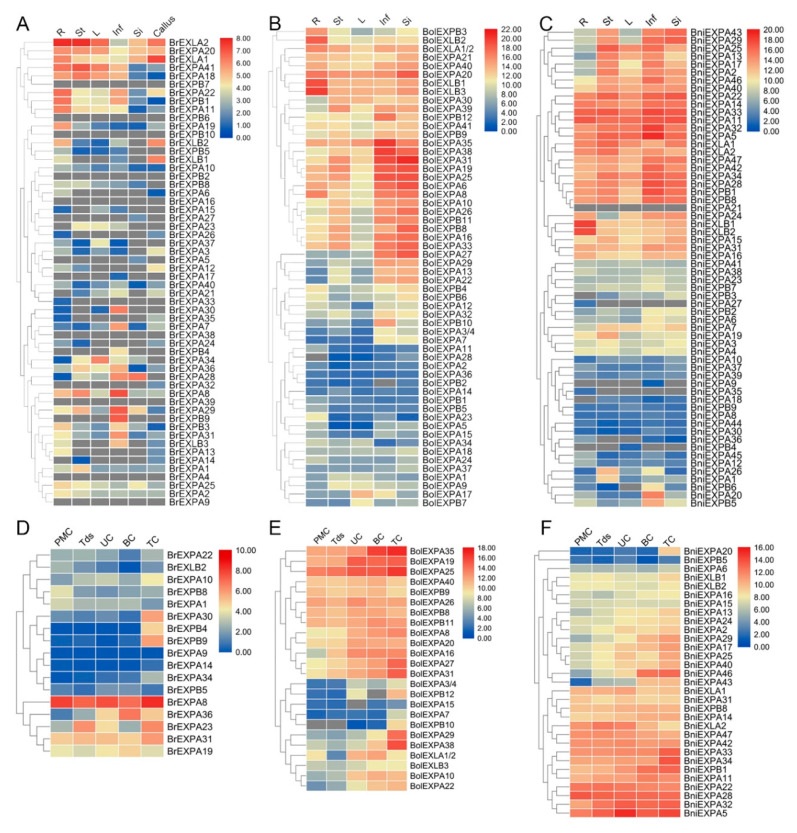
Hierarchical clustering and heat map generated by TBtools showing the expression levels of expansin genes in six or five tissues and different development stages of pollen in *B. rapa* (**A**,**D**), *B. oleracea* (**B**,**E**), and *B. nigra* (**C**,**F**). The scale bars represent relative expression level. The grey box designates undetectable expression. R roots, St stems, L leaves, Si siliques, Inf inflorescences, PMC pollen mother cell, Tds tetrads, UC uninuclear pollen, BC binucleate pollen, TC trinucleate pollen.

RNA-seq data were used to investigate the tissue-specific expression profile of *BrEXPs* in six tissues (root, stem, leaf, inflorescences, silique, and callus). However, the lack of RNA-Seq data for nine expansin genes (*BrEXPB2*/*6*/*7*, *BrEXPA4*/*9*/*10*/*16*/*38*/*39*) indicated that these genes may express only at specific developmental stages (e.g., *BrEXPA9* can be detected at the pollen development stage) or under special conditions (Figure 10A). Simultaneously, the expression patterns of 58 *BolEXPs* and 60 *BniEXPs* were studied by quantitative reverse transcription polymerase chain reaction (qRT-PCR) in five major tissues, including roots, stems, leaves, inflorescences and siliques. A total of 58 *BolEXPs* and 59 *BniEXPs* can be detected in at least one tissue (except *BniEXPA21*) (Figure 10B,C). Through the analysis of relevant data, the expression levels of expansin genes were related to various tissues, and the expression pattern of each expansin gene was different. In *B. rapa*, 35.7% of expansins were constitutively expressed in all six tissues. Meanwhile, due to the high precision of qRT-PCR, 73.2% and 85% of expansin genes were expressed in these five tissues in *B. oleracea* and *B. nigra*, but the level of expression varied. According to these results, we speculated that expansin genes play important roles in multiple development stages of *B. rapa*, *B. oleracea*, and *B. nigra*. Most of expansin genes appeared to be predominantly expressed in certain tissues. We found that 50% of *BrEXPs*, 55.4% of *BolEXPs*, and 58.3% of *BniEXPs* had peak expressions in a specific tissue. This result demonstrated that expansin genes had diverged, and they relaxed cell walls in corresponding organs.

Seventeen *BrEXPs*, 24 *BolEXPs*, and 31 *BniEXPs* had high expression levels in inflorescences and were used to study potential roles in the reproductive development of *B. rapa*, *B. oleracea*, and *B. nigra* (Figure 10A–C). Therefore, we used our previous RNA-Seq data of ‘*Bcajh97-01A*/*B*’ GMS line of *B. rapa* [40] and qRT-PCR method for *B. oleracea* and *B. nigra* to analyze their potential roles in pollen development. Through the study of *BolEXP* and *BniEXP* expression in five different stages of floral buds (pollen mother cell, tetrad, uninucleate pollen, binucleate pollen, and trinucleate pollen), we found that except for high expression levels of *BolEXPA7*/*10*/*27*/*29*/*31*/*38*, *BolEXPB12*, and *BniEXPA20*/*43*/*46* in mature pollen, other expansin genes were more uniformly expressed at different stages of pollen development (Figure 10E,F). For *B. rapa*, *BrEXPA8* was highly expressed at all stages, whereas *BrEXPA30* and *BrEXPB4*/*9* were prominent at the mature pollen stage (Figure 10D).

**Figure 11:**
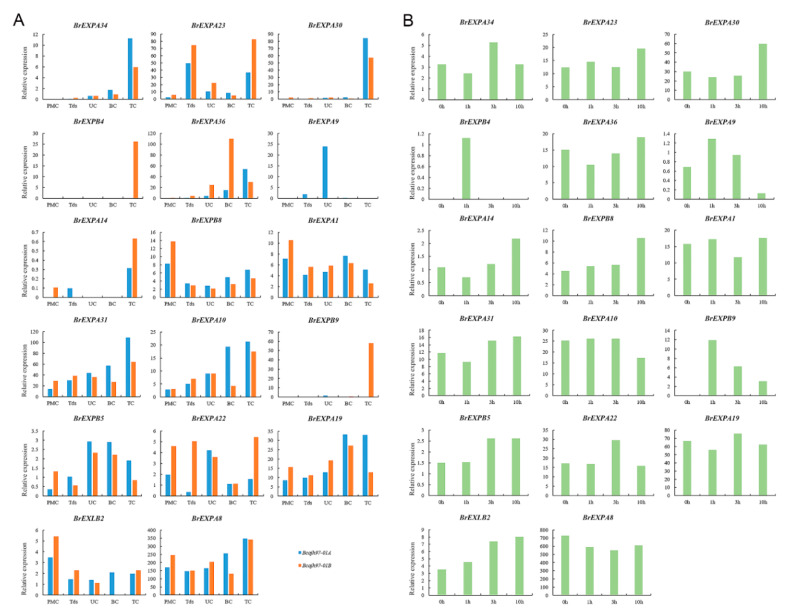
Expression profile analyses of representative expansin genes by RNA-Seq data in five development stages of pollen in ‘*Bcajh97-01A*/*B*’ (**A**) and pollinated pistils at 0, 1, 3, and 10 h after pollination (**B**). *PMC* pollen mother cell, *Tds* tetrads, *UC* uninuclear pollen, *BC* binucleate pollen, *TC* trinucleate pollen.

When comparing the expression levels of expansin genes in five pollen development stages of the fertile line *‘Bcajh97-01B’* and the sterile line *‘Bcajh97-01A’*, we found that the expression levels of *BrEXPA1*/*22*/*23* and *BrEXLB2* in the sterile line were lower than those in the fertile line during the tetrad period, suggesting that these genes had crucial roles in early anther development. Simultaneously, nine genes were expressed in all five stages, implying their continued roles in pollen development. Particularly, *BrEXPA8* was highly expressed during these periods, but the difference between fertile and sterile lines was not significant. However, *BrEXPB4*/*9* and *BrEXPA14*/*22* had high expression levels in the mature pollen stage of the fertile line but were comparatively low or even not expressed in the sterile line. Generally, genes that were highly expressed in mature pollen could continue to be expressed during pollination and fertilizations (Figure 11A). At 0, 1, 3, and 10 h after pollination, the expression levels of *BrEXPs* in pistil were observed. Particularly, for two copies of *AtEXPB5*, *BrEXPB4* and *BrEXPB9*, the expression level of *BrEXPB9* in the mature pollen stage and pollination and fertilization was higher than that of *BrEXPB4*. Hence, *BrEXPB4* was in the process of gradual degradation (Figure 11B).

## 3. Discussion

### 3.1. Scale of Expansin Families after WGT in B. rapa, B. oleracea, and B. nigra

The ancestor of diploid *Brassica* species and *A. thaliana* lineages diverged approximately 20 MYA. Subsequently, a WGT event occurred in the *Brassica* ancestor approximately 16 MYA. As the WGT of the *Brassica* ancestor, expansin genes in the *A. thaliana* genome might have triplicated orthologous copies in *B. rapa*, *B. oleracea* and *B. nigra* [30]. However, the total number of expansins in the three basic species of *Brassica* did not triple that of *A. thaliana* suggesting that expansin genes experienced loss and functional differentiation after WGT. The rapid loss of paralogs in genome-wide duplication might be due to the imbalance issue of gene dosage [41,42,43].

Recent research studies assumed that 60–90% of angiosperms underwent duplication events [44,45]. The size of a genome was expanded by duplication and provided a hotbed for the emergence of new gene functions. In duplication, several genes are likely to be retained as duplicates because of the need for certain functions in a particular organism [46]. These genes can follow one of three functional outcomes, including gene loss, neo-functionalization, and sub-functionalization [47]. Generally, the emergence of these new gene functions helped plants in adapting to the changing environment. Hence, they would not become extinct.

Of the three principal evolutionary patterns, segmental duplication, tandem duplication, and transposition [48], the expansion of expansin families in *B. rapa* and *B. nigra* seemed to be achieved mainly by segmental duplication (62.5% in *B. rapa*, 50.0% in *B. nigra*). Meanwhile, tandem duplication seemed to play only a minor role. Approximately 12.5% of expansin genes in *B. rapa*, 22.4% in *B. oleracea* and 15.0% in *B. nigra* were distributed in tandem arrays. However, in *B. oleracea*, the proportions of the segmental and tandem duplications were similar. According to a previous research, expansin families in different species showed species-specific expansion patterns. For instance, segmental duplication seemed to be the predominant form of expansin families in most dicots, such as *A. thaliana*, *B. rapa*, *B. nigra*, and soybean [25]. Inversely, tandem duplication is popular in monocots, such as rice and *Triticum aestivum* [5,35]. Intriguingly, for the expansin family, we observed that both segmental and tandem duplication played even roles in *B. oleracea*.

### 3.2. Conservative and Large Family Size of EXLB in Three Basic Species of Brassica

The EXLB subfamily is much larger in soybean than in *A. thaliana* and rice [25]. By comparing the size of expansin families in different species, we found that the size of EXLB subfamilies in monocotyledons was the smallest, accounting for 0%–1.79%, and the proportion of cruciferous plants was in the range of 2.78%–5.36%. Meanwhile, the EXLB subfamilies of Solanaceae were larger, reaching 10.5%–16.67% (Figure 1, Table 3). The different scales of the EXLB subfamilies suggested that they changed with the evolution of different families, but divergences among species in the same family were small. For the EXLB subfamilies in *A. thaliana* and three basic species of *Brassica*, we found that the pI values, length of proteins, and signal peptides were conserved, and they had the smallest ω values, suggesting that their sequences were the most conserved. The larger scales of EXLB subfamilies in the three species of *Brassica* might reflect adaptations to certain functions or environments. Hence, EXLB members have special functions in growth and development; for example, recent studies provided evidence to establish their functions in responding to abiotic stresses [15,49] and improving phosphorus acquisition [50].

### 3.3. Promoter Divergence is Closely Related to the Coding Sequence Evolution of Expansin Genes in Three Basic Species of Brassica

Although promoters can control gene expression at appropriate times, places, and levels and are related to phenotypic evolution [51], little is known about their own evolutionary divergences and their relationship to the evolution of coding sequences of specific gene families. Therefore, we applied *SharMot* [52], which effectively quantified differences between promoters by finding regions of high local similarity but did not consider order, direction, or spacing, to resolve whether or not the divergences of the promoters are correlated with the evolutionary rates of the coding sequences [53,54,55]. The method was also used to reveal the formation of pseudogenes in *A. thaliana* [56], similar trends in the upstream regions to the coding sequences of H3K27me3 [57], and relationship between promoters and protein sequences of the PG family in *B. rapa* [58].

In the present study, *SharMot* was used to explore evolutionary dynamics between upstream 500 bp sequences and sequences encoding the expansin genes of the orthologs and paralogs among *A. thaliana* and the three species of *Brassica*. We detected weak but significant positive correlations between *d_SM_* and *Ka*/*Ks* in orthologs of the three *Brassica* species (Figure 9). On the basis of the *Ka*/*Ks* results, expansin genes in the three species were under purification selection. According to a previous study [52], if *Ka*/*Ks* reflected the action of purifying selection, then a correlation in promoter and protein divergence indicated that the selective consequences of a deleterious mutation in either the promoter or protein coding sequence of a given gene were similar. However, this correlation was not found in paralogs. This result may be related to the selection of *B. rapa*, *B. oleracea*, and *B. nigra* after the common ancestor of *Brassica* diverged, leading to the departure of the promoter evolutionary pathway of the expansin genes to some extent. The distribution of *d_SM_* values also supported differences among four subfamilies (Figure 8).

### 3.4. Speculative Roles of Expansin Genes during Reproductive Development

*EXPB* genes are particularly numerous and abundantly expressed in grass, but the size of EXPB subfamilies is small in dicotyledons [59]. In the present study, we also found similar conclusion by comparing the size of expansin families of different species (Figure 1, Table 3). *EXPB* genes were previously thought to be members of pollen allergens and had cell wall-relaxing activity in herbaceous plants [59,60]. This phenomenon is explained by the fact that scales of EXPB subfamilies were much larger in monocots than in dicots. A recent study showed that Zea m 1 (EXPB1 of maize) and orthologous group-1 pollen allergens in other grasses were abundant in pollen and they could promote pollen tubes to enter the stigma and style by softening the maternal cell walls. The decreased expression levels of the EXPBs in maize can cause pollen accumulation, resulting in the poor dispersal of pollen when the anther is cracked, and pollen tubes cannot enter the corn whisker easily [61]. This result was also validated in a maize transgenic line that silenced all members of the EXPB subfamily [62]. However, we do not believe that the EXPB subfamily plays no role or play a minor role in the dicotyledon development. A tobacco EXPB subfamily gene, *PPAL*, was specifically expressed in the stigma secretion area and epidermal layer of the placenta, and expression levels increased during pollination and fertilization, indicating that it participated in the penetration of the pollen tube into the stigma [63]. The analysis of the expression profile of *A. thaliana* also showed that *AtEXPA4* and *AtEXPB5* are strongly expressed in dried pollen grains, swollen pollen, and pollen tubes. However, further research on their biological functions is needed [64].

Transcript abundance at specific times and organs is an important factor in characterizing the corresponding proteins required for subsequent developmental processes [65]. Our analysis of expansin gene expression in *B. rapa*, *B. oleracea*, and *B. nigra* indicated that they played indispensable roles in pollen development. This process was essential for plant reproduction, and significant implications for the selection of excellent vegetable varieties were observed. By analyzing the expression data, we found that the expression levels of 30.4% of *BrEXPs* (17 of 56), 44.8% of *BolEXPs* (26 of 58) and 51.7% of *BniEXPs* (31 of 60) in inflorescence were higher than those in other organs, suggesting the important roles of expansin genes in reproductive development (Figure 10D–F). Furthermore, we explored the potential roles of expansin genes during pollen development through the specific expression profiles of these *BrEXPs* at different flower bud stages and periods after pollination. Among them, two expansin genes (*BrEXPB4* and *BrEXPB9*) possessed similar expression patterns and showed significant differences in the fertile and sterile lines (Figure 11A). These expansin genes were also homologous genes of *AtEXPB5*, which maintained high expression level in pollen grains, suggesting that they had analogical biological functions in pollen development. Meanwhile, the expression level of *BrEXPB4* was lower than that of *BrEXPB9*, which demonstrated that *BrEXPB4* is in a stage of degradation. However, another *A. thaliana* gene, *AtEXPA4*, which was highly expressed in pollen grains, had two orthologous genes in *B. rapa*, namely *BrEXPA19* and *BrEXPA22*, which showed different expression patterns (Figure 11B). This result indicates that since the divergence of *A. thaliana* and *B. rapa*, orthologous genes gradually evolved different functions under the pressure of selection. Nonetheless, evidence in expression data alone was insufficient to clarify their functions during reproductive development. Hence, additional works are needed to provide evidence for this view.

## 4. Materials and Methods

### 4.1. Identification and Physicochemical Property Predictions of Expansin Family Members in Three Basic Diploid Species of Brassica

To identify all expansin gene family members in *B. rapa*, *B. oleracea*, and *B. nigra*, we performed a three-step analysis. First, the 36 protein sequences of the *A. thaliana* expansin family [66] were downloaded from the TAIR website (http://www.arabidopsis.org/) and compared with the sequences of *B. rapa* (genome v1.5), *B. oleracea* (genome v1.1), and *B. nigra* (genome v1.1) (BRAD, http://brassicadb.org/brad/) to obtain the first round of the candidates. Second, Pfam (http://www.sanger.ac.uk/Software/Pfam/), Simple Modular Architecture Research Tool (SMART, http://smart.embl-heidelberg.de/smart/batch.pl), and CDD/SPARCLE (https://www.ncbi.nlm.nih. gov/Structure/bwrpsb/bwrpsb.cgi) were used to confirm whether each predicted sequence was an expansin family member, sharing DPBB_1 (PF03330) and Pollen_allerg_1 (PF01357). Finally, the DNA sequences of candidates with missing domains and abnormal lengths, together with their 5 kb flanking regions of both sides, were analyzed and reannotated using FGENESH (http://linux1.softberry.com/berry.phtml?topic=fgenesh&group=programs&subgroup=gfind). The expansin genes of the three basic diploid species were nominated according to *A. thaliana* [6] and followed the order in which they appeared on chromosomes.

The computation of the pI values and molecular weights were carried through the Compute pI/Mw tool in ExPASy (http://web.expasy.org/compute_pi/), and signal peptide sequences were analyzed by SignalP 5.0 Server (http://www.cbs.dtu.dk/services/SignalP/).

### 4.2. Phylogenetic Genetic Tree Construction and Structural Analysis

A data file containing all the information from target genes (including the locations on the chromosomes, genomic sequences, coding sequences, protein sequences, and 1500 bp of the nucleotide sequences upstream of the translation initiation codon) was extracted from the files of *B. rapa*, *B. oleracea*, and *B. nigra*, which were downloaded from BRAD using the tools of TBtools [67].

Multiple sequence alignment was analyzed by MUSCLE in MEGA 6.0 software. According to amino acid sequence alignment, a phylogenetic tree was constructed by the maximum likelihood algorithm containing the protein sequences of AtEXPs and Br/Bol/BniEXPs. WAG + G (the WAG model + the “Gamma Distributed” option) was selected as the most suitable model through the model selection tool of MEGA 6.0. Other parameters were also set to the bootstrap replications of 1000 and “partial deletion” option. Finally, the visualization and beautification of the phylogenetic tree was accomplished by iTOL online tool (https://itol.embl.de/).

The composition and position of exons and introns for expansin genes of the four species were obtained by comparing the predicted coding sequences with their corresponding DNA sequences using TBtools. The online program MEME (http://meme-suite.org/) was used to detect eight conserved motifs contained for each gene with optimum motif width of ≥6 and ≤200 [68]. Furthermore, their conserved domains and relative positions on sequences were obtained from Pfam (http://www.sanger.ac.uk/Software/Pfam/). Lastly, the above results were visualized by TBtools.

### 4.3. Chromosome Location, Synteny and Retained Rate Analysis

The chromosomal locations of expansins in the three species of *Brassica* were retrieved from the genome data and mapped to chromosomes by TBtools with the Amazing Gene Location program.

For synteny analysis between *A. thaliana* and the three species, we used the Quick MCScanX Wrapper program of TBtools, which can be used in the Windows environment without any command line. Moreover, the results were visualized by the Amazing Super Circos tool.

Retention rates were calculated using the term “locus” instead of “gene” according to a previous study, which eliminated the disturbance of tandem duplication after WGT [33].

### 4.4. Evolution Analysis of Coding Sequences and Promoters

To understand the evolutionary dynamics of the expansin gene coding sequences, we performed comparisons from two dimensions, that is, horizontal and vertical. On the one hand, ω ratios (*Ka*/*Ks*), which represented selective pressure for different subfamilies in the four species were calculated separately using branch models of CODEML in PAML [69,70], and *p*-values were obtained from the R Project. Afterward, the ω ratios of every subgroup in these species were calculated using the branch models of EasyCodeML, which was a wrapper tool that provided a user-friendly graphical interface for using CODEML [71]. Two a priori assumptions were considered: the one-ratio model assumed that all branches had the same ω-parameter, whereas the free-ratio model allowed different branch values. To verify which model was best for the data, we applied likelihood ratio tests by comparing twice the difference in log-likelihood values between the pairs of models using a χ^2^ distribution. On the other hand, the Simple *Ka*/*Ks* Calculator program in TBtools was used to estimate *Ks*, *Ka*, and *Ka*/*Ks* ratios between homologous expansin gene pairs.

The *d_SM_* scores were used to evaluate the divergence between the upstream sequences of each homologous gene pair, which were obtained by *SharMot* software. By definition, *d_SM_* is the fraction of both sequences that do not contain a region of significant local similarity by specific criteria. For example, a *d_SM_* of 0 indicated a complete sharing of motifs between the sequences, whereas a *d_SM_* of 1.0 indicated the absence of shared motifs [52]. According to previous studies, we chose to analyze upstream 500 bp sequences from the start of translation, and determined that the optimal minimum length of a conservative upstream sequence was 18 bp [57,58,72]. SPSS 23.0 was used to calculate descriptive and inferential statistics and GraphPad Prism 5.01 was used to display the statistical results.

### 4.5. Expression Analysis of Expansin Family Members

The RNA-seq data of *B. rapa* were obtained from expression profile database (https://www.ebi.ac.uk/gxa/plant/experiments) and used to analyze the expression levels of the expansin genes in six tissues, including root, stem, leaf, inflorescences, silique, and callus [73].

Chinese cabbage (*B. rapa* subsp. *chinensis* cv. Aijiaohuang), ‘*Bcajh97-01A*/*B*’, a genic male sterile (GMS) sister line was used in this study. The sterile line ‘*Bcajh97-01A*’ is a male meiotic cytokinesis mutant without mature pollen, which is the only difference between ‘Bcajh97-01A’ and the fertile line ‘*Bcajh97-01B*’ [74]. Therefore, our previous RNA-Seq data on ‘*Bcajh97-01A*/*B*’ was ideal for studying expansin gene expression levels during pollen development, including five different flower bud stages (pollen mother cell, tetrad, uninuclear pollen, binucleate pollen, and trinucleate pollen stages) and pistils after pollination at 0, 1, 3, and 10 h [40].

However, given that the RNA-Seq data of *B. nigra* and *B. oleracea* are not available at present, the expression levels of expansin genes were measured using qRT-PCR. *B. nigra* (*B. nigra* cv. 1411-02) and *B. oleracea* (*B. oleracea* cv. Jingfengyihao) were kindly provided by Dr. Xiaolin Yu (Institute of Vegetable Science, Zhejiang University, Hangzhou, China) and grown in a 22 ± 1 °C growth chamber under long-day conditions (16 h light/8 h dark). The total RNA was extracted from five tissues including roots, stems, leaves, inflorescences, and siliques, and five different flower bud stages. To ensure the specificities, we designed the primers of each *BniEXP* and *BolEXP* by Primer Premier 5.0, and *UBC10* genes in *B. nigra* and *B. oleracea* were chosen as reference genes (Table S1) [58,75]. qRT-PCR was performed as described by [76]. For each sample, three biological replicates were conducted with three technical replicates, and the results were calculated using the 2^−ΔΔCt^ method [77].

## 5. Conclusions

Through the analyses and discussion of the above results, we can draw the following four conclusions. Firstly, through the construction of phylogenetic tree of the three basic species of *Brassica* and *A. thaliana*, we identified 56, 58 and 60 expansin genes in *B. rapa*, *B. oleracea* and *B. nigra*, and they were divided into four subfamilies. Each subfamily was divided into different subgroups, and there were 16 subgroups in total. Proper classification of gene family members facilitates exploration of evolution, expression, and function. Secondly, based on the analyses of the physicochemical properties, gene structures and conserved domains of expansin genes in the three basic *Brassica* species, and combined with previous studies, a brief model of an expansin family including typical elements of each subfamily was established (Figure 4). In particular, we noticed that EXLB subfamilies in *B. rapa*, *B. oleracea* and *B. nigra* were more conservative than other subfamilies. Further synteny analyses showed that the expansion of expansin families in these species was mainly due to segmental duplication rather than tandem duplication. Thirdly, the results of evolutionary rates in different clusters indicated that expansin families of the three basic species of *Brassica* were in the state of purification selection. In addition, by exploring the relationship between the promoter sequences and coding sequences of orthologous gene pairs, it showed that there were significant but weak positive correlations between upstream regulatory sequences and coding sequences of orthologs. Finally, studies on the expression patterns of *B. rapa*, *B. oleracea*, and *B. nigra* demonstrated that expansin genes had indispensable roles in most processes of plant growth and development, especially in reproductive development. Because EXPB subfamily proteins are abundant in the pollen of monocotyledons, they have received widespread attention in reproductive development, especially in the development of pollen. Although the scale of EXPB subfamilies was reduced in dicotyledons, similar gene functions may still be retained, such as *BrEXPB4* and *BrEXPB9*, whereas their specific biological functions should be further explored.

## Figures and Tables

**Table 1:** Numbers of expansin genes in different plants.

Species	EXPA	EXPB	EXLA	EXLB	Total	References
**Algae**						
*Micrasterias denticulata*					4′EXP’	[22]
**Moss**						
*Physcomitrella patens*	28	7	0	0	34	[23]
**Fern**						
*Selaginella moellendorffi*	15	2	0	0	17	[34]
**Gymnosperm**						
*Pinus taeda*	6	1	1	1	9	[5]
**Angiosperms**						
**Monocotyledon**						
Rice	33	18	4	1	56	[5]
Maize	36	48	4	0	88	[24]
*Triticum aestivum*	52	42	4	0	98	[35]
**Dicotyledon**						
**Cruciferae**						
*Arabidopsis thaliana*	26	6	3	1	36	[5]
*Brassica rapa*	41	10	2	3	56	
*Brassica oleracea*	41	12	2	3	58	
*Brassica nigra*	47	9	2	2	60	
**Solanaceae**						
Tobacco	36	6	3	7	52	[26]
Tomato	25	8	1	4	38	[36]
Potato	24	5	1	6	36	[18]
**Leguminosae**						
Soybean	49	9	2	15	75	[25]
Salicaceae						
Poplar	27	3	2	4	36	[37]
**Amborellaceae**						
*Amborella trichopoda*	17	2	1	2	22	[38]
**Vitaceae**						
Grape	20	4	1	4	29	[39]
**Rosaceae**						
Apple	34	1	2	4	41	[27]

**Table 2:** Detection of selection for expansin genes using branch model of PAML in the four subfamilies.

Species	Model	Estimates of Parameters	ln *L*	*LRT p*-Value
*Arabidopsis thaliana*	One-ratio	ω0 = 0.137 for all subfamilies	−13843.914525	0.4417919
	Free-ratio	ω1 = 999.000 for EXPA	−13840.170755	
		ω2 = 0.439 for EXPB		
		ω3 = 0.124 for EXLA		
		ω4 = 0.045 for EXLB		
*Brassica rapa*	One-ratio	ω0 = 0.118 for all subfamilies	−13413.618539	0.2491653
	Free-ratio	ω1 = 999.000 for EXPA	−13408.224099	
		ω2 = 999.000 for EXPB		
		ω3 = 0.225 for EXLA		
		ω4 = 0.005 for EXLB		
*Brassica oleracea*	One-ratio	ω0 = 0.107 for all subfamilies	−7890.958102	0.7815311
	Free-ratio	ω1 = 999.000 for EXPA	−7889.207635	
		ω2 = 999.000 for EXPB		
		ω3 = 0.122 for EXLA		
		ω4 = 0.070 for EXLB		
*Brassica nigra*	One-ratio	ω0 = 0.125 for all clades	−11781.483673	0.9198657
	Free-ratio	ω1 = 999.000 for EXPA	−11780.551263	
		ω2 = 999.000 for EXPB		
		ω3 = 0.154 for EXLA		

ln *L*, the log-likelihood value; *LRT p*-value, the likelihood ratio test *p*-value.

**Table 3:** The mean evolutionary rates (*Ka, Ks* and *Ka/Ks*) and promoter divergences (*d_SM_*) of different subfamilies’ orthologous expansin genes between *A. thaliana* and *B. rapa*, *A. thaliana* and *B. oleracea*, and *A. thaliana* and *B. nigra*.

		EXPA	EXPB	EXLA	EXLB
*A. thaliana* vs. *B. rapa*	*Ka*	0.085(0.067) ^a^	0.106 (0.055)	0.072 (0.006)	0.059 (0.005)
	*Ks*	0.508(0.114)	0.589 (0.114)	0.493 (0.034)	0.338 (0.077)
	*Ka*/*Ks*	0.149(0.090) ^a^	0.174 (0.070)	0.146 (0.002)	0.183 (0.051)
	*d_SM_*	0.600(0.286)	0.747 (0.315)	0.803 (0.062)	0.720 (0.076)
*A. thaliana* vs. *B. oleracea*	*Ka*	0.053(0.026) ^b^	0.112 (0.055)	0.076 (0.002)	0.069 (0.015)
	*Ks*	0.487(0.093)	0.568 (0.059)	0.465 (0.073)	0.329 (0.032)
	*Ka*/*Ks*	0.116(0.057) ^b^	0.202 (0.083)	0.165 (0.021)	0.212 (0.058)
	*d_SM_*	0.592(0.256)	0.762 (0.304)	0.775 (0.101)	0.694 (0.059)
*A. thaliana* vs. *B. nigra*	*Ka*	0.048(0.019) ^b^	0.127 (0.063)	0.076 (0.013)	0.061 (0.010)
	*Ks*	0.486(0.100)	0.548 (0.068)	0.478 (0.069)	0.340 (0.039)
	*Ka*/*Ks*	0.114(0.059) ^b^	0.207 (0.083)	0.160 (0.003)	0.182 (0.049)
	*d_SM_*	0.573(0.285)	0.692 (0.285)	0.738 (0.151)	0.672 (0.100)

Standard deviation is shown in parentheses. Lower-case letters (^a^ and ^b^) indicate significant differences (*p* < 0.05, Tukey test).

## Supplementary Materials

Supplementary materials can be found at https://www.mdpi.com/1422-0067/21/10/3424/s1. Table S1. Primers used in qRT-PCR analysis of expansin genes in *B. nigra* and *B. oleracea*. Table S2. Identification and physicochemical property predictions of expansin genes in *A. thaliana*, *B. rapa*, *B. oleracea*, and *B. nigra* and nomenclature for the three basic species of *Brassica*. Table S3. Evolutionary rate analyses of orthologous expansin genes and upstream 500 bp promoters between *A. thaliana* and *B. rapa*, *A. thaliana* and *B. oleracea*, and *A. thaliana* and *B. nigra*. Table S4. Evolutionary rate analyses of paralogous expansin genes and upstream 500 bp promoters in *B. rapa*, *B. oleracea*, and *B. nigra*, respectively. Table S5. Detection of selection for expansin genes using branch model of EasyCodeML in different subgroups. Table S6. The mean evolutionary rates (*Ka*, *Ks* and *Ka*/*Ks*) and promoter divergences (*d_SM_*) of different subfamilies’ paralogous expansin genes in *B. rapa*, *B. oleracea*, and *B. nigra*. Figure S1. Genomic distribution of expansin genes on chromosomes of *A. thaliana*, *B. rapa*, *B. oleracea*, and *B. nigra*. Figure S2. The *Ka*, *Ks*, *Ka*/*Ks* distributions of paralogous expansin gene pairs in *B. rapa*, *B. oleracea*, and *B. nigra*. Figure S3. The *d_SM_* distributions of paralogous expansin gene pairs in *B. rapa*, *B. oleracea*, and *B. nigra*.

## Abbreviations

*BrEXPs*	*B. rapa* expansin genes
*BolEXPs*	*B. oleracea* expansin genes
*BniEXPs*	*B. nigra* expansin genes
GMS	genic male sterile
*Ka*	nonsynonymous substitution rate
*Ks*	synonymous substitution rate
Mw	molecular weights
MYA	million years ago
*PGs*	polygalacturonases genes
pIs	isoelectric points
qRT-PCR	quantitative reverse transcription polymerase chain reaction
WGT	whole genome triplication

## References

[1] Sanchez-Rodriguez C., Rubio-Somoza I., Sibout R., Persson S. (2010). Phytohormones and the cell wall in Arabidopsis during seedling growth. Trends Plant Sci..

[2] Cosgrove D.J. (2015). Plant expansins: Diversity and interactions with plant cell walls. Curr. Opin. Plant Biol..

[3] Cosgrove D.J. (2005). Growth of the plant cell wall. Nat. Rev. Mol. Cell Biol..

[4] Cosgrove D.J., Li L.C., Cho H.T., Hoffmann-Benning S., Moore R.C., Blecker D. (2002). The growing world of expansins. Plant Cell Physiol..

[5] Sampedro J., Cosgrove D.J. (2005). The expansin superfamily. Genome Biol..

[6] Kende H., Bradford K., Brummell D., Cho H.T., Cosgrove D., Fleming A., Gehring C., Lee Y., McQueen-Mason S., Rose J. (2004). Nomenclature for members of the expansin superfamily of genes and proteins. Plant Mol. Biol..

[7] Sanchez-Montesino R., Bouza-Morcillo L., Marquez J., Ghita M., Duran-Nebreda S., Gomez L., Holdsworth M.J., Bassel G., Onate-Sanchez L. (2019). A Regulatory Module Controlling GA-Mediated Endosperm Cell Expansion Is Critical for Seed Germination in Arabidopsis. Mol. Plant.

[8] Ren H., Wen L.Z., Guo Y.H., Yu Y.Y., Sun C.H., Fan H.M., Ma F.F., Zheng C.S. (2019). Expressional and Functional Verification of the Involvement of CmEXPA4 in Chrysanthemum Root Development. J. Plant Growth Regul..

[9] Stamm P., Topham A.T., Mukhtar N.K., Jackson M.D., Tome D.F., Beynon J.L., Bassel G.W. (2017). The Transcription Factor ATHB5 Affects GA-Mediated Plasticity in Hypocotyl Cell Growth during Seed Germination. Plant Physiol..

[10] Kuluev B.R., Knyazev A.V., Nikonorov Y.M., Chemeris A.V. (2013). Role of the expansin genesNtEXPA1andNtEXPA4in the regulation of cell extension during tobacco leaf growth. Russ. J. Genet..

[11] Han Y.C., Kuang J.F., Chen J.Y., Liu X.C., Xiao Y.Y., Fu C.C., Wang J.N., Wu K.Q., Lu W.J. (2016). Banana Transcription Factor MaERF11 Recruits Histone Deacetylase MaHDA1 and Represses the Expression of MaACO1 and Expansins during Fruit Ripening. Plant Physiol..

[12] Lou Y., Zhou H.S., Han Y., Zeng Q.Y., Zhu J., Yang Z.N. (2018). Positive regulation of AMS by TDF1 and the formation of a TDF1-AMS complex are required for anther development in *Arabidopsis thaliana*. New Phytol..

[13] Valdivia E.R., Wu Y., Li L.C., Cosgrove D.J., Stephenson A.G. (2007). A group-1 grass pollen allergen influences the outcome of pollen competition in maize. PLoS ONE.

[14] Zhang S., Wang J., Chen G., Ye X., Zhang L., Zhu S., Yuan L., Hou J., Wang C. (2019). Functional analysis of a MYB transcription factor BrTDF1 in the tapetum development of Wucai (*Brassica rapa* ssp.). Sci. Hortic..

[15] Krishnamurthy P., Muthusamy M., Kim J.A., Jeong M.J., Lee S.I. (2019). *Brassica rapa* expansin-like B1 gene (BrEXLB1) regulate growth and development in transgenic Arabidopsis and elicits response to abiotic stresses. J. Plant Biochem. Biotechnol..

[16] Liu Y.P., Zhang L., Hao W.F., Zhang L., Liu Y., Chen L.Q. (2019). Expression of Two alpha-Type Expansins from *Ammopiptanthus nanus* in *Arabidopsis thaliana* Enhance Tolerance to Cold and Drought Stresses. Int. J. Mol. Sci..

[17] Han Z., Liu Y., Deng X., Liu D., Liu Y., Hu Y., Yan Y. (2019). Genome-wide identification and expression analysis of expansin gene family in common wheat (*Triticum aestivum* L.). BMC Genom..

[18] Chen Y., Zhang B., Li C., Lei C., Kong C., Yang Y., Gong M. (2019). A comprehensive expression analysis of the expansin gene family in potato (*Solanum tuberosum*) discloses stress-responsive expansin-like B genes for drought and heat tolerances. PLoS ONE.

[19] Otulak-Koziel K., Koziel E., Lockhart B.E.L., Bujarski J.J. (2020). The Expression of Potato Expansin A3 (StEXPA3) and Extensin4 (StEXT4) Genes with Distribution of StEXPAs and HRGPs-Extensin Changes as an Effect of Cell Wall Rebuilding in Two Types of PVY(NTN)-Solanum tuberosum Interactions. Viruses.

[20] Fernando G.J., Sebastian L., Heike T., Britt-Louise L., Thomas K., David G., Edgar M., Mark V., Pathology S.E.I.J.M.P. (2018). Massive up-regulation of LBD transcription factors and EXPANSINs highlights the regulatory programs of rhizomania disease. Mol. Plant Pathol..

[21] Tan J., Wang M., Shi Z., Miao X. (2018). OsEXPA10 mediates the balance between growth and resistance to biotic stress in rice. Plant Cell Rep..

[22] Vannerum K., Huysman M.J., De Rycke R., Vuylsteke M., Leliaert F., Pollier J., Lutz-Meindl U., Gillard J., De Veylder L., Goossens A. (2011). Transcriptional analysis of cell growth and morphogenesis in the unicellular green alga Micrasterias (Streptophyta), with emphasis on the role of expansin. BMC Plant Biol..

[23] Carey R.E., Cosgrove D.J. (2007). Portrait of the expansin superfamily in Physcomitrella patens: Comparisons with angiosperm expansins. Ann. Bot..

[24] Zhang W., Yan H., Chen W., Liu J., Jiang C., Jiang H., Zhu S., Cheng B. (2014). Genome-wide identification and characterization of maize expansin genes expressed in endosperm. Mol. Genet. Genom..

[25] Zhu Y., Wu N., Song W., Yin G., Qin Y., Yan Y., Hu Y. (2014). Soybean (*Glycine max*) expansin gene superfamily origins: Segmental and tandem duplication events followed by divergent selection among subfamilies. BMC Plant Biol..

[26] Ding A., Marowa P., Kong Y. (2016). Genome-wide identification of the expansin gene family in tobacco (Nicotiana tabacum). Mol. Genet. Genom..

[27] Zhang S., Xu R., Gao Z., Chen C., Jiang Z., Shu H.J.M.G., Genomics (2014). A genome-wide analysis of the expansin genes in Malus×Domestica. Mol. Genet. Genom..

[28] Krishnamurthy P., Hong J.K., Kim J.A., Jeong M.J., Lee Y.H., Lee S.I. (2015). Genome-wide analysis of the expansin gene superfamily reveals *Brassica rapa*-specific evolutionary dynamics upon whole genome triplication. Mol. Genet. Genom..

[29] Town C.D., Cheung F., Maiti R., Crabtree J., Haas B.J., Wortman J.R., Hine E.E., Althoff R., Arbogast T.S., Tallon L.J. (2006). Comparative genomics of *Brassica oleracea* and *Arabidopsis thaliana* reveal gene loss, fragmentation, and dispersal after polyploidy. Plant Cell.

[30] Yu J., Tehrim S., Zhang F., Tong C., Huang J., Cheng X., Dong C., Zhou Y., Qin R., Hua W. (2014). Genome-wide comparative analysis of NBS-encoding genes between Brassica species and *Arabidopsis thaliana*. BMC Genom..

[31] Yang T.J., Kim J.S., Kwon S.J., Lim K.B., Cell B.-S.P.J.P. (2006). Sequence-level analysis of the diploidization process in the triplicated FLOWERING LOCUS C region of *Brassica rapa*. Plant Cell.

[32] Lysak M.A., Koch M.A., Pecinka A., Schubert I. (2005). Chromosome triplication found across the tribe Brassiceae. Genome Res..

[33] Wu P., Shao Z.Q., Wu X.Z., Wang Q., Wang B., Chen J.Q., Hang Y.Y., Xue J.Y. (2014). Loss/retention and evolution of NBS-encoding genes upon whole genome triplication of *Brassica rapa*. Gene.

[34] Carey R.E., Hepler N.K., Cosgrove D.J. (2013). Selaginella moellendorffii has a reduced and highly conserved expansin superfamily with genes more closely related to angiosperms than to bryophytes. BMC Plant Biol..

[35] Li N., Pu Y., Gong Y., Yu Y., Ding H. (2016). Genomic location and expression analysis of expansin gene family reveals the evolutionary and functional significance in *Triticum aestivum*. Genes Genom..

[36] Lu Y., Liu L., Wang X., Han Z., Ouyang B., Zhang J., Li H. (2016). Genome-wide identification and expression analysis of the expansin gene family in tomato. Mol. Genet. Genom..

[37] Sampedro J., Carey R.E., Cosgrove D.J. (2006). Genome histories clarify evolution of the expansin superfamily: New insights from the poplar genome and pine ESTs. J. Plant Res..

[38] Seader V.H., Thornsberry J.M., Carey R.E. (2016). Utility of the Amborella trichopoda expansin superfamily in elucidating the history of angiosperm expansins. J. Plant Res..

[39] Dal Santo S., Vannozzi A., Tornielli G.B., Fasoli M., Venturini L., Pezzotti M., Zenoni S. (2013). Genome-wide analysis of the expansin gene superfamily reveals grapevine-specific structural and functional characteristics. PLoS ONE.

[40] Shen X., Xu L., Liu Y., Dong H., Zhou D., Zhang Y., Lin S., Cao J., Huang L. (2019). Comparative transcriptome analysis and ChIP-sequencing reveals stage-specific gene expression and regulation profiles associated with pollen wall formation in *Brassica rapa*. BMC Genom..

[41] Freeling M. (2008). The evolutionary position of subfunctionalization, downgraded. Genome Dyn..

[42] Freeling M. (2009). Bias in plant gene content following different sorts of duplication: Tandem, whole-genome, segmental, or by transposition. Annu Rev. Plant Biol..

[43] Papp B., Pal C., Hurst L.D. (2003). Dosage sensitivity and the evolution of gene families in yeast. Nature.

[44] Schmutz J., Cannon S.B., Schlueter J., Ma J., Mitros T., Nelson W., Hyten D.L., Song Q., Thelen J.J., Cheng J. (2010). Genome sequence of the palaeopolyploid soybean. Nature.

[45] Moore R.C., Purugganan M.D. (2003). The early stages of duplicate gene evolution. Proc. Natl. Acad. Sci. USA.

[46] Lynch M., Conery J.S. (2000). The evolutionary fate and consequences of duplicate genes. Science.

[47] Force A., Lynch M., Pickett F.B., Amores A., Postlethwait J.J.G. (1999). Preservation of Duplicate Genes by Complementary, Degenerative Mutations. Genetics.

[48] Cannon S.B., Mitra A., Baumgarten A., Young N.D., May G. (2004). The roles of segmental and tandem gene duplication in the evolution of large gene families in *Arabidopsis thaliana*. BMC Plant Biol..

[49] Guimaraes L.A., Mota A.P.Z., Araujo A.C.G., de Alencar Figueiredo L.F.D., Pereira B.M., de Passos Saraiva M.A., Silva R.B., Danchin E.G.J., Guimaraes P.M., Brasileiro A.C.M. (2017). Genome-wide analysis of expansin superfamily in wild Arachis discloses a stress-responsive expansin-like B gene. Plant Mol.Biol..

[50] Wang B., Du H., Li W.L., Li X.H., Zhang C.Y. (2019). GmEXLB1, a Soybean Expansin-Like B Gene, Alters Root Architecture to Improve Phosphorus Acquisition in Arabidopsis. Front. Plant Sci..

[51] Wray G.A., Hahn M.W., Abouheif E., Balhoff J.P., Pizer M., Rockman M.V., Romano L.A. (2003). The evolution of transcriptional regulation in eukaryotes. Mol. Biol. Evol..

[52] Castillo-Davis C.I., Hartl D.L., Achaz G. (2004). cis-Regulatory and protein evolution in orthologous and duplicate genes. Genome Res..

[53] Haberer G., Hindemitt T., Meyers B.C., Mayer K.F. (2004). Transcriptional similarities, dissimilarities, and conservation of cis-elements in duplicated genes of Arabidopsis. Plant Physiol..

[54] Casneuf T., De Bodt S., Raes J., Maere S., Van de Peer Y. (2006). Nonrandom divergence of gene expression following gene and genome duplications in the flowering plant Arabidopsis thaliana. Genome Biol..

[55] Ganko E.W., Meyers B.C., Vision T.J. (2007). Divergence in expression between duplicated genes in Arabidopsis. Mol. Biol. Evol..

[56] Yang L., Takuno S., Waters E.R., Gaut B.S. (2011). Lowly expressed genes in Arabidopsis thaliana bear the signature of possible pseudogenization by promoter degradation. Mol. Biol. Evol..

[57] Berke L., Sanchez-Perez G.F., Snel B. (2012). Contribution of the epigenetic mark H3K27me3 to functional divergence after whole genome duplication in Arabidopsis. Genome Biol..

[58] Liang Y., Yu Y., Shen X., Dong H., Lyu M., Xu L., Ma Z., Liu T., Cao J. (2015). Dissecting the complex molecular evolution and expression of polygalacturonase gene family in *Brassica rapa* ssp. chinensis. Plant Mol. Biol..

[59] Cosgrove D.J. (2000). New genes and new biological roles for expansins. Curr. Opin. Plant Biol..

[60] Cosgrove D.J. (2000). Loosening of plant cell walls by expansins. Nature.

[61] Cosgrove D.J., Bedinger P., Durachko D.M. (1997). Group I allergens of grass pollen as cell wall-loosening agents. Proc. Natl. Acad. Sci. USA.

[62] Valdivia E.R., Stephenson A.G., Durachko D.M., Cosgrove D. (2009). Class B beta-expansins are needed for pollen separation and stigma penetration. Sex. Plant Reprod..

[63] Pezzotti M., Feron R., Mariani C. (2002). Pollination modulates expression of the PPAL gene, a pistil-specific beta-expansin. Plant Mol. Biol..

[64] Mollet J.C., Leroux C., Dardelle F., Lehner A. (2013). Cell Wall Composition, Biosynthesis and Remodeling during Pollen Tube Growth. Plants.

[65] Wu J., Peng Z., Liu S., He Y., Cheng L., Kong F., Wang J., Lu G. (2012). Genome-wide analysis of Aux/IAA gene family in Solanaceae species using tomato as a model. Mol. Genet. Genom..

[66] Lee Y., Choi D., Kende H. (2001). Expansins: Ever-expanding numbers and functions. Curr. Opin. Plant Biol..

[67] Chen C., Xia R., Chen H., He Y. (2018). TBtools, a Toolkit for Biologists integrating various HTS-data handling tools with a user-friendly interface. bioRxiv.

[68] Lin Y.X., Jiang H.Y., Chu Z.X., Tang X.L., Zhu S.W., Cheng B.J. (2011). Genome-wide identification, classification and analysis of heat shock transcription factor family in maize. BMC Genom..

[69] Yang Z. (1997). PAML: A program package for phylogenetic analysis by maximum likelihood. Comput. Appl. Biosci..

[70] Yang Z. (2007). PAML 4: Phylogenetic analysis by maximum likelihood. Mol. Biol. Evol..

[71] Gao F., Chen C., Arab D.A., Du Z., He Y., Ho S.Y.W. (2019). EasyCodeML: A visual tool for analysis of selection using CodeML. Ecol. Evol..

[72] Yang L., Gaut B.S. (2011). Factors that contribute to variation in evolutionary rate among Arabidopsis genes. Mol. Biol. Evol..

[73] Tong C., Wang X., Yu J., Wu J., Li W., Huang J., Dong C., Hua W., Liu S. (2013). Comprehensive analysis of RNA-seq data reveals the complexity of the transcriptome in *Brassica rapa*. BMC Genom..

[74] Huang L., Ye W.-Z., Liu T.-T., Cao J.-S. (2009). Characterization of the Male-sterile Line Bcajh97-01A/B and Identification of Candidate Genes for Genic Male Sterility in Chinese Cabbage-pak-choi. J. Am. Soc. Hortic. Sci..

[75] Lin S., Yue X., Miao Y., Yu Y., Dong H., Huang L., Cao J. (2018). The distinct functions of two classical arabinogalactan proteins BcMF8 and BcMF18 during pollen wall development in *Brassica campestris*. Plant J..

[76] Hu Z., Shen X., Xiang X., Cao J. (2019). Evolution of MIR159/319 genes in *Brassica campestris* and their function in pollen development. Plant Mol. Biol..

[77] Livak K.J., Schmittgen T.D. (2001). Analysis of relative gene expression data using real-time quantitative PCR and the 2(-Delta Delta C(T)) Method. Methods.

