# Supplementary material for: Complex Molecular Evolution and Expression of Expansin Gene Families in Three Basic Diploid Species of Brassica
Source: Int J Mol Sci. 2020 May 12;21(10):3424. doi: 10.3390/ijms21103424 (PMC7279145; doi:10.3390/ijms21103424)
Supplement: Supplementary file 1 [file ijms-21-03424-s001.zip › Supplementary data/Supplementary Figures S1-S3.pdf]

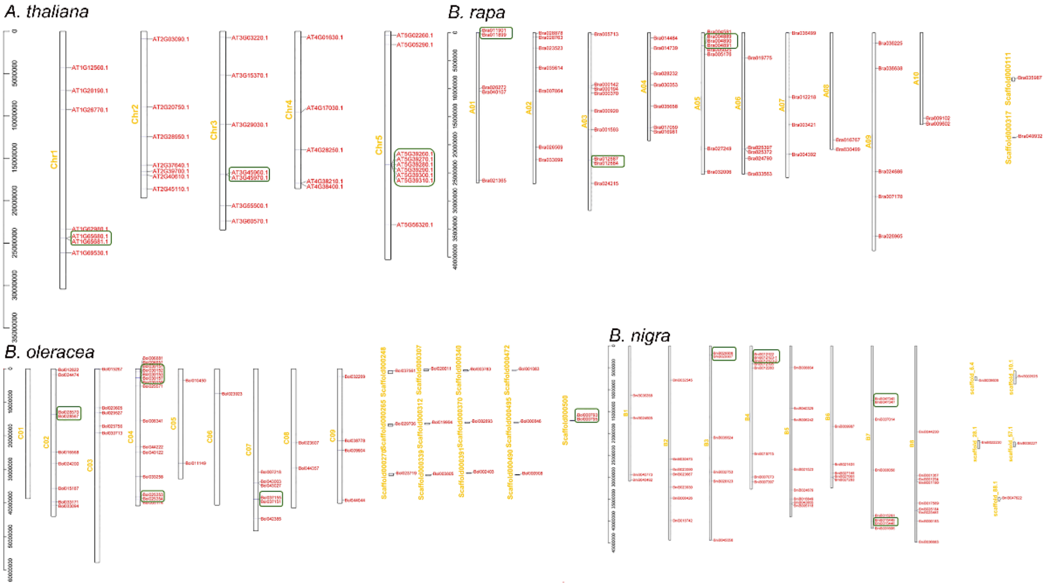

Fig. S1. Genomic distribution of expansin genes on chromosomes of *A. thaliana*, *B. rapa*, *B. oleracea*, and *B. nigra*. Green boxes stand for tandem genes in corresponding genomes. The chromosome numbers are demonstrated at left of each chromosome. The rulers on the left represent the length of the nucleotide sequences.

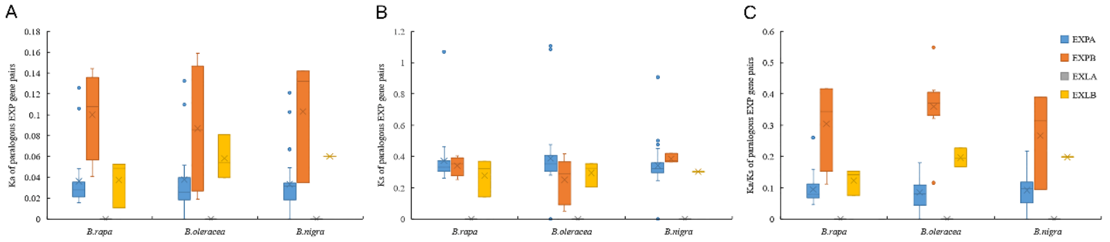

Fig. S2. The *Ka*, *Ks*, *Ka*/*Ks* distributions of paralogous expansin gene pairs in *B. rapa*, *B. oleracea*, and *B. nigra*. (A–C) *Box plots* of *Ka*, *Ks*, and *Ka*/*Ks* of orthologs. Vertical lines represent the range of values, boxes represent interquartile distances, and the colored line represents the median. The circle represents the abnormal values and the cross represents the average values.

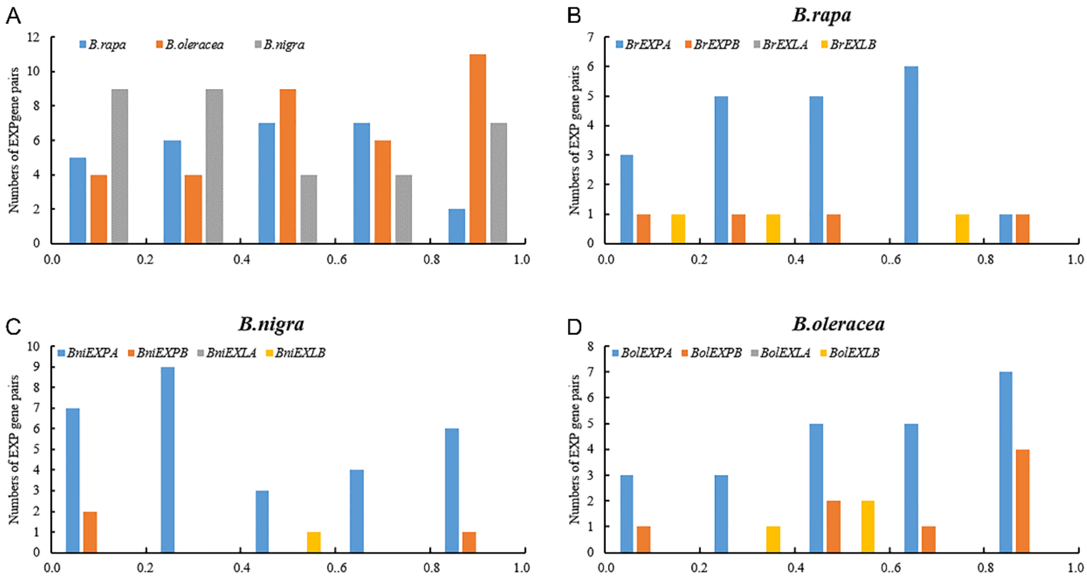

Fig. S3. The d_{SM} distributions of paralogous expansin gene pairs in *B. rapa*, *B. oleracea*, and *B. nigra.* (A) The d_{SM} distribution of expansin gene pairs in three basic species of *Brassica*. (B–D) The d_{SM} distribution of expansin gene pairs in four subfamilies of *B. rapa* (B), *B. oleracea* (C), and *B. nigra* (D). Paralogous gene pairs did not exist in the EXLA subfamilies of three *Brassica* subspecies.
