# Supplementary material for: Complex Molecular Evolution and Expression of Expansin Gene Families in Three Basic Diploid Species of Brassica
Source: Int J Mol Sci. 2020 May 12;21(10):3424. doi: 10.3390/ijms21103424 (PMC7279145; doi:10.3390/ijms21103424)
Supplement: Supplementary file 1 [file ijms-21-03424-s001.zip › Supplementary data/Supplementary Tables S5-S6.pdf]

Supporting Information Table S5. Detection of selection for expansin genes using branch model of EasyCodeML in different subgroups.

Species	Model	Estimates of parameters	ln *L*	*LRT p*-value
Arabidopsis thaliana	Model 0	ω0=0.137 for all subgroups	-13843.915	
	Two Ratio Model 2	ω1=0.058 for EXPA-I	-13843.201	0.232
		ω2=0.078 for EXPA-II	-13842.549	0.098
		ω3=0.287 for EXPA-III	-13843.588	0.419
		ω4=0.316 for EXPA-IV	-13842.971	0.170
		ω5=0.285 for EXPA-V	-13842.193	0.063
		ω6=0.372 for EXPA-VI	-13841.691	0.035
		ω7=0.005 for EXPA-VII	-13841.815	0.040
		ω8=0.004 for EXPA-VIII	-13842.144	0.060
		ω9=0.155 for EXPA-IX	-13843.889	0.823
		ω10=0.283 for EXPA-X	-13843.356	0.290
		ω11=0.034 for EXPA-XI	-13843.774	0.60
		ω12=0.002 for EXPA-XII	-13840.674	0.011
		ω13=0.001 for EXPB-I	-13842.554	0.099
		ω14=0.003 for EXPB-II	-13841.753	0.038
		ω15=0.087 for EXLA-I	-13843.770	0.590
		ω16=0.041 for EXLB-I	-13842.509	0.094
Brassica rapa	Model 0	ω0=0.118 for all subgroups	-13413.619	
	Two Ratio Model 2	ω1=0.234 for EXPA-I	-13413.318	0.438
		ω2=0.102 for EXPA-II	-13413.520	0.658

		ω3=0.343 for EXPA-III	-13412.862	0.219
		ω4=1.148 for EXPA-IV	-13410.874	0.019
		ω5=0.147 for EXPA-V	-13413.507	0.636
		ω6=0.421 for EXPA-VI	-13411.938	0.067
		ω7=0.102 for EXPA-VII	-13413.591	0.815
		ω8=0.196 for EXPA-VIII	-13413.212	0.367
		ω9=0.161 for EXPA-IX	-13413.530	0.674
		ω10=0.115 for EXPA-X	-13413.618	0.981
		ω11=0.400 for EXPA-XI	-13412.164	0.088
		ω12=0.205 for EXPA-XII	-13413.586	0.798
		ω13=0.001 for EXPB-I	-13411.777	0.055
		ω14=0.003 for EXPB-II	-13409.574	0.004
		ω15=0.345 for EXLA-I	-13413.223	0.374
		ω16=0.005 for EXLB-I	-13411.967	0.069
Brassica oleracea	Model 0	ω0=0.107 for all subgroups	-7890.958	
	Two Ratio Model 2	ω1=0.366 for EXPA-I	-7890.767	0.538
		ω2=0.098 for EXPA-II	-7890.945	0.871
		ω3=0.177 for EXPA-III	-7890.179	0.442
		ω4=0.948 for EXPA-IV	-7889.036	0.050
		ω5=0.022 for EXPA-V	-7890.170	0.209
		ω6=0.026 for EXPA-VI	-7889.862	0.139
		ω7=0.066 for EXPA-VII	-7890.870	0.675

		$\omega_8=0.037$ for EXPA-VIII	-7889.995	0.165
		$\omega_9=0.316$ for EXPA-IX	-7889.935	0.153
		$\omega_{10}=0.051$ for EXPA-X	-7890.813	0.591
		$\omega_{11}=1.468$ for EXPA-XI	-7890.265	0.239
		$\omega_{12}=0.243$ for EXPA-XII	-7890.429	0.304
		$\omega_{13}=0.058$ for EXPB-I	-7890.949	0.891
		$\omega_{14}=0.002$ for EXPB-II	-7889.389	0.077
		$\omega_{15}=0.121$ for EXLA-I	-7890.952	0.914
		$\omega_{16}=0.056$ for EXLB-I	-7890.769	0.539
Brassica nigra	Model 0	$\omega_0=0.125$ for all subgroups	-11781.484	
	Two Ratio Model 2	$\omega_1=2.510$ for EXPA-I	-11779.870	0.0724
		$\omega_2=2.510$ for EXPA-II	-11779.870	0.0724
		$\omega_3=0.250$ for EXPA-III	-11781.137	0.405
		$\omega_4=0.686$ for EXPA-IV	-11781.125	0.397
		$\omega_5=0.144$ for EXPA-V	-11781.444	0.777
		$\omega_6=0.125$ for EXPA-VI	-11781.484	0.998
		$\omega_7=0.028$ for EXPA-VII	-11780.648	0.196
		$\omega_8=0.132$ for EXPA-VIII	-11781.480	0.929
		$\omega_9=0.123$ for EXPA-IX	-11781.484	0.988
		$\omega_{10}=0.030$ for EXPA-X	-11781.311	0.557
		$\omega_{11}=1.195$ for EXPA-XI	-11777.456	0.005
		$\omega_{12}=1.019$ for EXPA-XII	-11781.280	0.523

	ω13=0.243 for EXPB-I	-11781.475	0.897
	ω14=0.070 for EXPB-II	-11781.403	0.688
	ω15=0.158 for EXLA-I	-11781.441	0.771
	ω16=0.128 for EXLB-I	-11781.483	0.974

ln *L* the log-likelihood value, *LRT* *p*-value the likelihood ratio test *p* value

Supporting Information Table S6. The mean evolutionary rates (*Ka*, *Ks* and *Ka*/*Ks*) and promoter divergences (d_{SM}) of different subfamilies' paralogous expansin genes in *B. rapa*, *B. oleracea*, and *B. nigra*.

		**EXPA**	**EXPB**	**EXLA**	**EXLB**
B. rapa* vs *B. rapa	*Ka*	0.027(0.008)	0.120(0.021)	N/A	0.037(0.023)
	Ks	0.336(0.048)	0.341(0.063)	N/A	0.346(0.035)
	Ka/*Ks*	0.086(0.030)	0.368(0.082)	N/A	0.147(0.007)
	d_{SM}	0.492(0.235)	0.436(0.364)	N/A	0.449(0.233)
B. oleracea* vs *B. oleracea	*Ka*	0.027(0.011)	0.123(0.036)	N/A	0.058(0.021)
	Ks	0.348(0.043)	0.315(0.097)	N/A	0.295(0.078)
	Ka/*Ks*	0.093(0.054)	0.369(0.030)	N/A	0.196(0.030)
	d_{SM}	0.607(0.310)	0.697(0.342)	N/A	0.456(0.112)
B. nigra* vs *B. nigra	*Ka*	0.027(0.009)	0.137(0.007)	N/A	0.060(N/A)
	Ks	0.322(0.044)	0.386(0.030)	N/A	0.303(N/A)
	Ka/*Ks*	0.096(0.046)	0.352(0.053)	N/A	0.198(N/A)
	d_{SM}	0.480(0.337)	0.342(0.398)	N/A	0.525(N/A)

Standard deviation is shown in parentheses
